# Integrator Complex Subunit 6 Regulates Biological Nature of Hepatocellular Carcinoma by Modulating Epithelial–Mesenchymal Transition

**DOI:** 10.3390/cimb47090733

**Published:** 2025-09-09

**Authors:** Sayaka Yonezawa, Keishi Kanno, Minami Shiozaki, Masanori Sugiyama, Masanori Ito

**Affiliations:** 1 Department of General Internal Medicine, Hiroshima University Hospital, 1-2-3 Kasumi, Minami-ku, Hiroshima 734-8551, Japan; sayaka22@hiroshima-u.ac.jp (S.Y.); beautifulwave.sio@gmail.com (M.S.); maito@hiroshima-u.ac.jp (M.I.); 2 Department of Probiotic Science for Preventive Medicine, Graduate School of Biomedical and Health Sciences, Hiroshima University, Hiroshima 734-8551, Japan; sugi@hiroshima-u.ac.jp

**Keywords:** hepatocellular carcinoma, integrator complex subunit 6, epithelial–mesenchymal transition, sorafenib, small activating RNA

## Abstract

Hepatocellular carcinoma (HCC) is a leading cause of cancer-related death worldwide, with limited therapeutic options and frequent resistance to treatment. The integrator complex subunit 6 (INTS6), a regulator of RNA polymerase II transcription, has emerged as a potential tumor suppressor that modulates Wnt/β-catenin signaling and epithelial–mesenchymal transition (EMT). This study aimed to clarify the role of INTS6 in EMT regulation in HCC and to explore the therapeutic potential of small activating RNA (saRNA)-mediated INTS6 induction. The Cancer Genome Atlas (TCGA) dataset was analyzed to assess the clinical relevance of INTS6 in HCC. Functional studies were conducted using a hepatoma cell line to determine the effects of INTS6 modulation on tumor behavior. Data analysis demonstrated that low INTS6 expression was associated with shorter disease-free survival and poorer prognosis in patients receiving conservative treatment. Experimental suppression of INTS6 increased mesenchymal marker expression, whereas saRNA-mediated induction suppressed these markers. Restoring INTS6 expression reduced cell migration, invasion, and proliferation through G1 cell-cycle arrest and enhanced sensitivity to sorafenib. These findings identify INTS6 as a promising therapeutic target in HCC. saRNA-mediated induction of INTS6 may provide a novel strategy, alone or in combination therapy, to overcome drug resistance and improve clinical outcomes.

## 1. Introduction

Hepatocellular carcinoma (HCC) is one of the leading causes of cancer-related mortality worldwide, with a poor prognosis largely due to late-stage diagnosis and limited therapeutic options [1,2]. The pathogenesis of HCC involves both genetic and epigenetic alterations that disrupt key signaling pathways [3]. Current treatment modalities include surgical resection, liver transplantation, and systemic therapies such as tyrosine kinase inhibitors and immunotherapies [1,4]. However, these conservative treatments often yield only modest survival benefits and are hindered by immune-related adverse reactions and drug resistance [5]. Recent research efforts have focused on identifying novel genetic targets and exploring advanced therapeutic approaches, including gene therapy, nanoparticles, and chimeric antigen receptor (CAR)-T-cell therapy [1,6].

Epithelial–mesenchymal transition (EMT) is a biological process in which epithelial cells lose polarity and adhesion and acquire migratory and invasive properties. In cancer, EMT drives metastasis, drug resistance, and tumor progression [7,8,9]. In HCC, EMT is regulated by pathways such as Wnt/β-catenin, TGF-β, and STAT3, with long non-coding RNAs (lncRNAs) also playing a role [10,11]. EMT-related gene signatures have been developed to predict prognosis and treatment response in HCC [10,12]. Targeting EMT and its associated pathways offers a promising strategy for improving HCC management.

The integrator complex is composed of at least 14 subunits and plays a critical role in the regulation of RNA polymerase II (RNAPII)-mediated transcription, RNA processing, and DNA repair [13,14]. Among its components, the integrator complex subunit 6 (INTS6) recruits protein phosphatase 2A (PP2A) to the chromatin, thereby modulating its activity and influencing transcriptional regulation [15]. Dysregulation of the PP2A–integrator–cyclin-dependent kinase 9 (CDK9) axis is commonly observed in various cancers and can arise from genetic alterations or overexpression of inhibitory molecules [15,16,17]. Several integrin subunits display aberrant expression profiles in HCC, indicating their potential utility as diagnostic and prognostic biomarkers [18]. Notably, INTS6 acts as a tumor suppressor by inhibiting the Wnt/β-catenin signaling pathway, thereby impeding HCC progression, whereas INTS8 enhances EMT via activation of the TGF-β pathway, promoting tumor aggressiveness [19,20]. Furthermore, INTS6 interacts with INTS3 to form a complex essential for the repair of DNA double-stranded breaks [21]. Collectively, these findings underscore the diverse roles of the individual integrator subunits in cancer biology, highlighting INTS6 as a promising therapeutic target in HCC.

Small activating RNAs (saRNAs) are emerging as promising therapeutic agents for cancer treatment. These short double-stranded RNAs can upregulate the expression of specific genes through RNA activation, providing a novel mechanism for gene regulation [22,23]. In this study, we aimed to explore the impact of saRNA-induced INTS6 expression on the malignant behavior of HCC cells, with a particular focus on its effects on EMT and drug sensitivity. Given their therapeutic potential, saRNAs, along with small interfering RNAs (siRNAs), are increasingly recognized as key components of nucleic acid-based medicine, offering significant promise for clinical drug development.

## 2. Materials and Methods

### 2.1. Open Data Analysis

The association between *INTS6* mRNA expression levels obtained from RNA sequencing and the clinical characteristics of HCC (*n* = 372) was analyzed using data from The Cancer Genome Atlas (TCGA) (https://www.cancer.gov/ccg/research/genome-sequencing/tcga (accessed on 13 February 2024)). The analysis was conducted using the open-source platform cBioPortal (https://www.cbioportal.org/ (accessed on 13 February 2024)), which enables the exploration of genomic data and their correlation with clinical features such as tumor stage, survival outcomes, and mutational profiles.

### 2.2. Cell Lines and Cell Culture

Huh7 cells, a human hepatoma cell line in which the absence of CTNNB1 mutations has been explicitly documented in previous reports [24], were obtained from the Japanese Collection of Research Bioresources Cell Bank, Osaka, Japan. The cells were cultured in Dulbecco’s Modified Eagle Medium (Wako, Tokyo, Japan) supplemented with 10% fetal bovine serum (Gibco, Waltham, MA, USA).

### 2.3. SaRNA/SiRNA Transfection

Human INTS6-specific saRNA was synthesized based on a previously described sequence [25,26]. The human -specific siRNA and a non-silencing negative control were purchased from Sigma-Aldrich (St. Louis, MO, USA). Sequences of the saRNAs and siRNAs used in this study are listed in Supplementary Table S1. siRNAs and saRNAs, at a concentration of 20 nM, were reverse-transfected using Lipofectamine^®^ RNAiMAX (Invitrogen, Carlsbad, CA, USA) and incubated for 24 h.

### 2.4. Gene Expression Analysis

RNA was isolated using the FastGene RNA Basic Kit (NIPPON Genetics Co., Ltd., Tokyo, Japan). Complementary DNA was synthesized from 1 µg of total RNA using ReverTra Ace^®^ (TOYOBO, Osaka, Japan). Specific primer sequences were designed using Primer 3 (https://bioinfo.ut.ee/primer3-0.4.0/ (accessed on 6 June 2025)), and are listed in Supplementary Table S2. Real-time PCR was conducted on a CFX Connect Real-Time System (BIO-RAD, Hercules, CA, USA) using THUNDERBIRD™ Next SYBR^®^ qPCR Mix (TOYOBO, Osaka, Japan).

### 2.5. Immunoblot Analysis

The cells were lysed with Radioimmunoprecipitation Assay Buffer and centrifuged at 10,000× *g* for 10 min. The resulting supernatant was subjected to immunoblot analysis using primary antibodies against INTS6 (GeneTex, Irvine, CA, USA), β-catenin, N-cadherin, vimentin, snail family transcriptional repressor 1 (SNAI1) (Proteintech, IL, USA), and glyceraldehyde-3-phosphate dehydrogenase (GAPDH) (Sigma-Aldrich, St. Louis, MO, USA).

### 2.6. Migration and Invasion Assay

Huh7 cells were seeded on cell-culture inserts with an 8-µm pore-sized membrane (BD Biosciences, Bedford, MA, USA). For the invasion assay, the membrane was precoated with 100 µL of Matrigel diluted to a final concentration of 200 µg/mL (Corning Incorporated, Corning, NY, USA), whereas no Matrigel coating was applied for the migration assay. After 72 h of incubation, the cells that passed through the membrane were fixed in 10% paraformaldehyde and stained with crystal violet.

### 2.7. Scratch Assay

Huh7 cells were seeded in 6-well plates and grown to confluence. A scratch was created using a sterile 200 µL pipette tip, and detached cells were removed by washing with PBS. Images of the scratched areas were captured at 0, 6, and 24 h, using an inverted microscope.

### 2.8. Cell-Cycle Analysis Using Flow Cytometry

Cell-cycle analysis was performed using the BD Pharmingen™ PI/RNase Staining Buffer (BD Biosciences, Franklin Lakes, NJ, USA) according to the manufacturer’s protocol. Briefly, cells were harvested, washed with PBS, fixed with 70% ethanol, and stained with PI/RNase Staining Buffer. Samples were incubated at 4 °C for 2 h. Stained cells were harvested, washed with 2% FBS-PBS, resuspended in PI/RNase Staining Buffer, and analyzed using the BD LSRFortessa™ X-20 flow cytometer (BD Biosciences), to determine the percentage of cells in each phase of the cell cycle (G0/G1, S, and G2/M).

### 2.9. Cell Proliferation Assay

Cell proliferation was evaluated using Cell-Counting Kit-8 (CCK-8) (Dojindo Laboratories, Kumamoto, Japan). Huh7 cells were transfected with saRNAs targeting INTS6 or the control for 24 h and seeded in 96-well plates for 11 days. At each time point, CCK-8 reagent was added, and the absorbance at 450 nm was measured using an iMark™ Microplate Reader (Bio-Rad, Hercules, CA, USA).

### 2.10. Brdu Incorporation Assay

Cell proliferation was also assessed using a 5-bromo-2′-deoxyuridine (BrdU) incorporation assay (BrdU Cell Proliferation Assay Kit, Abcam, Cambridge, UK). Three days after transfection, cells were seeded into 96-well plates. Twelve hours later, cells were treated as indicated and incubated with 10 μM BrdU for 12 h. Following fixation and DNA denaturation, an anti-BrdU antibody and an HRP-conjugated secondary antibody were applied sequentially. The signal was developed using TMB substrate, and the absorbance was measured at 450 nm with a microplate reader.

### 2.11. Assessment of Sorafenib Sensitivity in Huh7 Cells

To evaluate the effect of INTS6 expression on sorafenib sensitivity in Huh7 cells, the half-maximal inhibitory concentration (IC_50_) was determined. Huh7 cells were transfected with saRNAs targeting INTS6 or a non-targeting control. At 72 h post-transfection, cells were treated with increasing concentrations of sorafenib (0, 2.5, 5.0, 7.5, 10, and 50 μM) for 24 h. Cell viability was measured using the CCK-8, and IC_50_ values were calculated using dose–response curve fitting. To assess cytotoxic effects, lactate dehydrogenase (LDH) release into the culture supernatant was quantified at 24 h using an LDH Cytotoxicity Assay Kit (Dojindo Laboratories, Kumamoto, Japan). The LDH release was expressed as a percentage of the maximum LDH release in the control and normalized to that of the untreated controls. Apoptotic cell death was evaluated by fluorescence microscopy using a Leica Stellaris 5 confocal microscope (Leica Microsystems, Wetzlar, Germany) and by flow cytometry using an Annexin V-FITC Apoptosis Detection Kit (Nacalai Tesque, Inc., Kyoto, Japan), in accordance with the manufacturer’s protocol.

### 2.12. Statistical Analysis

All data are presented as mean ± standard deviation (SD). Statistical significance was determined using a two-tailed Student’s *t*-test, except for the open data analysis, where the log-rank test was applied. A *p*-value < 0.05 was considered statistically significant.

## 3. Results

### 3.1. Clinical Characteristics of INTS6 Expression in HCC

**Figure 1:**
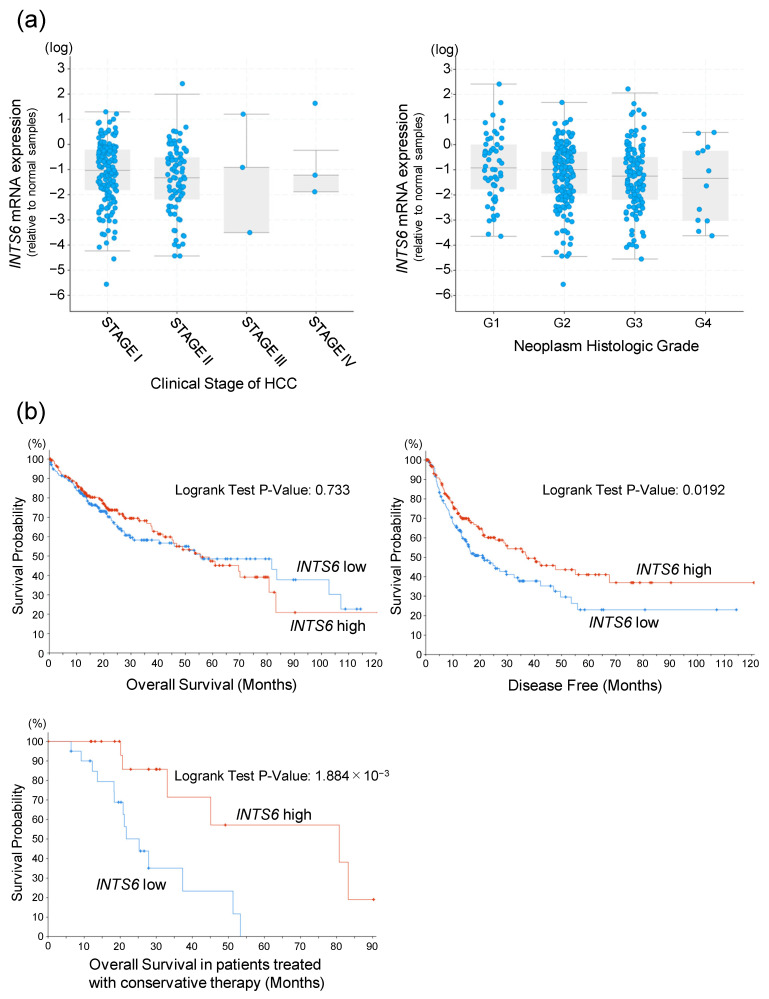
*INTS6* mRNA expression and clinical characteristics in hepatocellular carcinoma (HCC). Data were obtained from The Cancer Genome Atlas (TCGA) (*n* = 372) and analyzed using cBioPortal to explore correlations with clinical features. (**a**) *INTS6* mRNA expression levels (log scale) stratified by clinical stage (I–IV) and histologic grade (G1–G4). Scatter plots are presented as the mean ± standard deviation. (**b**) Survival analysis of patients stratified according to *INTS6* expression. Overall survival (OS) (log-rank test, *p* = 0.733). Disease-free survival (DFS) (log-rank test, *p* = 0.0192). OS of conservatively treated patients (*n* = 41) (log-rank test, *p* = 1.884 × 10^−3^). Red and blue lines indicate high and low *INTS6* expression levels, respectively.

Clinical data for HCC were obtained from TCGA, which provides information on the clinical stage and neoplasm histologic grade for a total of 372 patients with HCC. The analysis indicated that *INTS6* mRNA expression in tumors was lower than that in the corresponding normal tissues in most cases. Its expression level did not correlate with the clinical stage at diagnosis; however, a trend of decreased *INTS6* expression was observed in patients with higher histologic grades (Figure 1a). To evaluate the clinical significance of *INTS6*, the patients were stratified into two subgroups based on the median *INTS6* expression level. While overall survival was unaffected by *INTS6* expression, disease-free survival was significantly shorter in the low *INTS6* expression group (*p* = 0.0192), suggesting a higher risk of recurrence. Additionally, an analysis of 41 patients treated with conservative therapies, including chemotherapy and radiotherapy, revealed that those with low *INTS6* expression had a significantly poorer prognosis (*p* = 1.884 × 10^−3^), indicating potential resistance to conservative treatment (Figure 1b).

### 3.2. INTS6 Regulates EMT in HCC Cells

**Figure 2:**
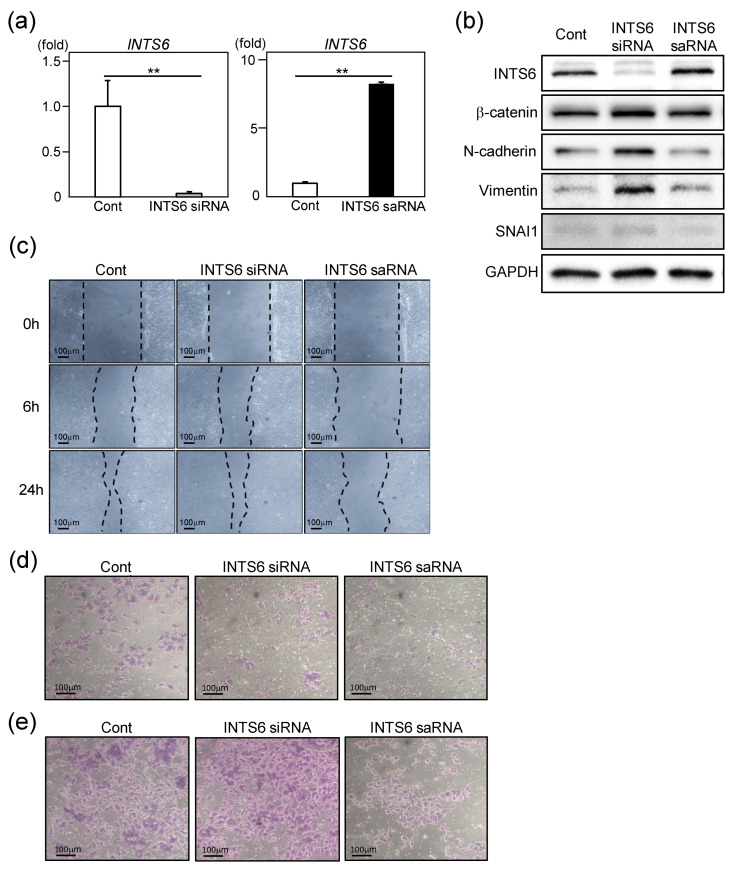
Effects of INTS6 modulation on epithelial–mesenchymal transition (EMT) and cell motility. (**a**) qRT-PCR analysis of INTS6 expression following siRNA-mediated knockdown (siRNA) or saRNA-mediated activation (saRNA). Relative *INTS6* mRNA expression levels compared to the control (cont), normalized to *GAPDH*. ** *p* < 0.01. (**b**) Immunoblot analysis of INTS6 and EMT-related markers, including β-catenin, N-cadherin, vimentin, and SNAI1. GAPDH was used as a loading control. (**c**) Wound-healing assay showing cell migration at 0 h, 6 h, and 24 h after scratching in control, INTS6 siRNA, and INTS6 saRNA-transfected cells. Dashed lines indicate the wound margins. (**d**,**e**) Invasion and migration assays demonstrating the effects of INTS6 modulation. Representative images of the invasion and migration assay for siRNA- and saRNA-transfected cells after 72 h of incubation.

Because EMT plays a crucial role in HCC progression, metastasis, and therapeutic resistance, we hypothesized that INTS6 regulates EMT in HCC cells [27]. To test this hypothesis, we examined the influence of INTS6 expression on EMT phenotypes in HCC cells using siRNA and saRNA (Figure 2a). As shown in Figure 2b, INTS6 knockdown led to the upregulation of β-catenin, one of master regulators of EMT, along with mesenchymal markers such as N-cadherin, vimentin, and SNAI1. These observations contrast with those observed upon INTS6 induction by saRNA.

To assess the impact of INTS6 expression on HCC cell migration, we performed a scratch assay, which revealed a modest decrease in wound-closure capacity following INTS6 knockdown using siRNA. Conversely, the migration of HCC cells was markedly reduced when INTS6 expression was upregulated using saRNA (Figure 2c). INTS6 suppression consistently increased the number of invading and migrating cells, whereas INTS6 induction reduced this number (Figure 2d,e). These findings suggest that INTS6 is involved in EMT and cellular migratory behavior in HCC cells, supporting the clinical observation that INTS6 regulates the malignant phenotype of HCC (Figure 1).

### 3.3. Upregulation of INTS6 Reduces HCC Cell Proliferation

**Figure 3:**
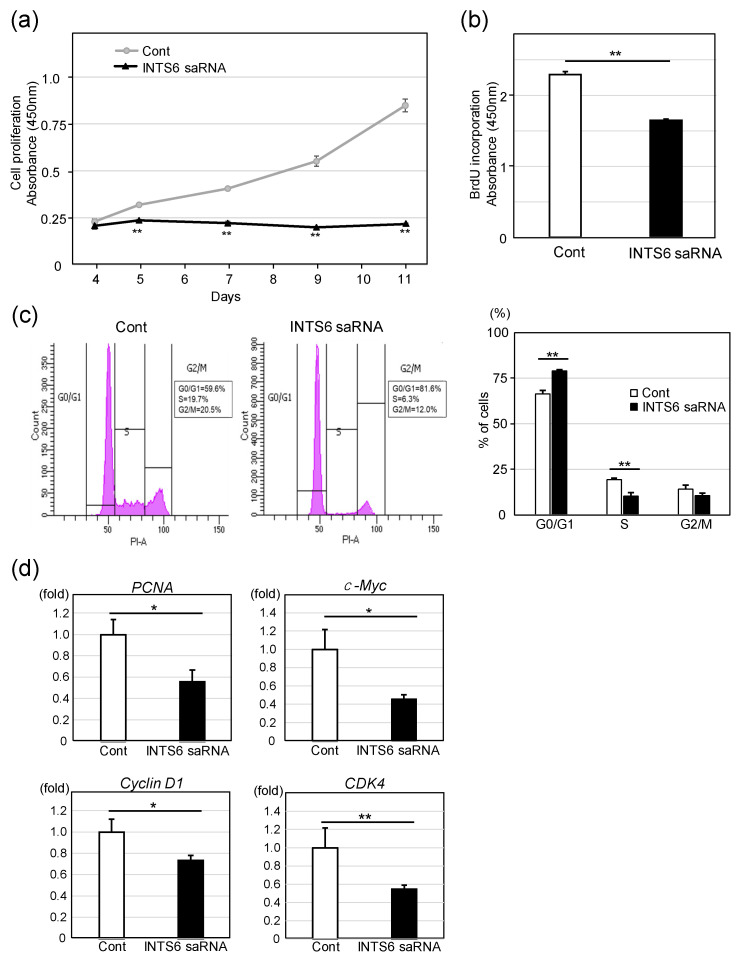
Induction of INTS6 inhibits cell proliferation. (**a**) Cell proliferation was assessed using the CCK-8 assay in Huh7 cells transfected with INTS6-targeting saRNA or control. Absorbance at 450 nm was measured over 11 days, showing significantly reduced proliferation in the INTS6 saRNA group. ** *p* < 0.01. Data are mean ± SE (*n* = 3). (**b**) 5-Bromo-2′-deoxyuridine (BrdU) incorporation assay was performed three days after transfection, demonstrating a significant decrease in DNA synthesis in INTS6-induced cells. ** *p* < 0.01. (**c**) Flow cytometry analysis of cell-cycle distribution, indicating G0/G1 arrest and reduced S-phase in INTS6 saRNA-transfected cells, indicating inhibited proliferation. ** *p* < 0.01. (**d**) The expression level of cell cycle-related genes such as *PCNA*, *c-MYC*, *Cyclin D1*, and *CDK4* was examined by real-time PCR following INTS6 induction (*n* = 5). * *p* < 0.05, ** *p* < 0.01.

Next, we investigated the effect of INTS6 overexpression on the proliferative potential of HCC cells, with a focus on its implications for therapeutic applications. Interestingly, INTS6 induction by saRNA markedly inhibited cell proliferation (Figure 3a). Consistently, BrdU incorporation was reduced by approximately 30% in Huh7 cells transfected with INTS6 saRNA relative to control cells, indicating a significant suppression of DNA synthesis (Figure 3b). To elucidate the mechanisms underlying INTS6-mediated cell growth reduction, we analyzed cell-cycle distribution in Huh7 cells using PI staining. As shown in Figure 3b, INTS6 induction significantly increased the proportion of cells in the G0/G1 phase and decreased the proportion in the S phase compared to those in the control, suggesting that INTS6 induces G1 cell cycle arrest. Consistent with these findings, the expression levels of cell-cycle-related genes such as *PCNA*, *c*-*MYC*, *Cyclin D1*, and *CDK4* were downregulated following INTS6 induction.

### 3.4. INTS6 Induction Enhances the Sensitivity of HCC Cells to Sorafenib

**Figure 4:**
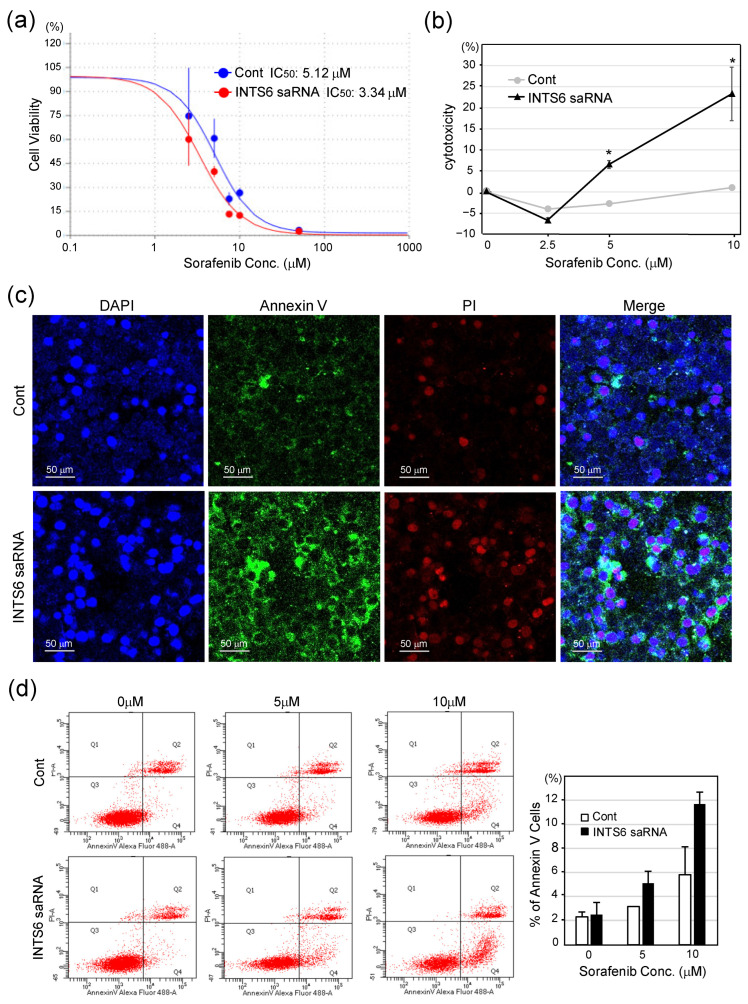
INTS6 saRNA enhances the sorafenib sensitivity of the HCC cell. (**a**) Dose–response curves of Huh7 cells treated with sorafenib following transfection with control or INTS6-targeting saRNA. Cell viability was assessed using a CCK-8 assay after 24 h of sorafenib exposure. IC_50_ values were calculated as 5.12 µM for control and 3.34 µM for INTS6 saRNA. (**b**) Lactate dehydrogenase (LDH) assay assessing cytotoxicity in Huh7 cells transfected with INTS6 saRNA or control and treated with increasing concentrations of sorafenib. Data are presented as mean ± SE (*n* = 3). INTS6 saRNA significantly increased sorafenib-induced cytotoxicity. * *p* < 0.05. (**c**) Apoptosis analysis by Annexin V/PI staining. Representative fluorescence microscopy images of Huh7 cells transfected with control or INTS6 saRNA, treated with 5 µM of sorafenib, followed by Annexin V-FITC staining, propidium iodide (PI), and DAPI. (**d**) Quantification of Annexin V–positive cells shown in (**c**). INTS6 induction significantly increased the percentage of apoptotic cells upon sorafenib treatment.

To evaluate the effect of INTS6 induction on sorafenib sensitivity, we compared the viability of Huh7 cells transfected with INTS6-targeting saRNA or control RNA, followed by sorafenib treatment. As shown in Figure 4a, INTS6-induced cells exhibited a significant reduction in viability in response to increasing concentrations of sorafenib compared to the control. The IC_50_ of sorafenib was decreased from 5.12 μM in control to 3.34 μM in INTS6-induced cells, indicating enhanced drug sensitivity. Consistently, LDH release was significantly higher in cells transfected with INTS6 saRNA than that in the control group (Figure 4b). Apoptosis was assessed using Annexin V and PI staining. Fluorescence microscopy revealed a substantial increase in the number of Annexin V-positive cells in the INTS6 saRNA group after sorafenib treatment (Figure 4c). Quantitative analysis confirmed that the percentage of apoptotic cells was significantly higher in the INTS6-induced group, particularly when 10 μM of sorafenib was used (Figure 4d). These findings suggest that INTS6 induction enhances the sensitization of HCC cells to sorafenib.

## 4. Discussion

Systemic therapies for advanced-stage HCC offer limited survival benefits, and recent advances such as immune checkpoint inhibitors and targeted agents remain constrained by underlying liver cirrhosis and immune-related adverse events [1,2,3,4,28,29,30]. Given these limitations, RNA-based therapies are being actively explored as promising alternatives and they have demonstrated encouraging antitumor activity in preclinical models and early clinical trials. siRNAs targeting cyclin E induce apoptosis and tumor regression, whereas VEGF- or integrin-targeted siRNAs delivered via nanoparticle systems delay tumor progression, reduce macrophage infiltration, and improve survival [31,32,33]. Furthermore, saRNA targeting CEBPA has shown 2.5–3-fold mRNA upregulation, growth inhibition, and reduced tumor burden [34,35]. Delivery systems such as liposomes and dendrimers also improve liver function markers and support early clinical translation [36]. Collectively, both siRNA and saRNA strategies modulate key genetic pathways to suppress tumor growth and enhance liver function in HCC. Building on the recent advances in RNA-based therapeutics, this study aimed to investigate the potential of INTS6-targeting saRNAs as a novel treatment strategy for HCC. Given that INTS6 expression is frequently downregulated in tumor tissues, particular attention has been directed toward EMT regulation as a key mechanism underlying tumor progression and therapeutic resistance.

Analysis of the TCGA database revealed that *INTS6* expression was decreased in most HCC tissues compared to adjacent normal tissues. This finding is consistent with previous reports showing reduced INTS6 expression in liver, breast, lung, and prostate cancers [18,37,38,39], suggesting a potential role in tumor suppression. While overall survival did not differ significantly between the high- and low-expression groups, disease-free survival was shorter in the low-expression group than in the high-expression group. Among the patients receiving conservative treatment, those with low *INTS6* expression had a significantly poorer prognosis. Taken together, these findings suggest that reduced INTS6 expression is associated with greater malignant potential and increased risk of recurrence.

One potential explanation for the association between low INTS6 expression and malignant nature in HCC is the involvement of EMT, a biological process closely linked to cancer progression, metastasis, and therapeutic resistance [7,8]. Per a previous study that used HCC cell lines, INTS6 suppresses the Wnt/β-catenin signaling pathway and induces changes in the expression of EMT-related genes [19]. However, the impact of INTS6 on cellular characteristics associated with EMT has not yet been thoroughly investigated. The present study clearly demonstrated that the induction of INTS6 expression resulted in the downregulation of mesenchymal markers and a concomitant reduction in the migratory and invasive capacities of HCC cells. Moreover, given the established role of EMT in drug sensitivity, we found that INTS6 upregulation enhanced cellular sensitivity to sorafenib [7,8,40]. These findings suggest that the restoration of INTS6 expression may reduce the malignant potential of HCC cells and improve their therapeutic responsiveness, highlighting its potential as a novel target for HCC treatment. While cancer is recognized as a multifactorial disease involving numerous signaling pathways and gene networks, our results also indicate that INTS6 may function as an important regulatory component within this complex landscape, contributing to broader mechanisms of tumor progression and drug resistance.

Our findings demonstrated that INTS6 induction significantly suppressed HCC cell proliferation by promoting G1 phase arrest, highlighting its potential role as a tumor suppressor. This observation is consistent with previous reports showing that exogenous re-expression of INTS6 in prostate cancer cells inhibits colony formation through a similar mechanism, accompanied by the upregulation of genes related to the Wnt signaling pathway [41]. In addition, CDK9—a cyclin-dependent kinase that is essential for transcriptional elongation via the phosphorylation of RNAPII—has been implicated as a downstream effector of INTS6. Notably, the loss of INTS6 disrupts the recruitment of PP2A to CDK9 in leukemia and unspecified solid tumors, resulting in sustained CDK9 activity and resistance to CDK9 inhibitors [14]. These findings underscore the multifaceted tumor-suppressive functions of INTS6, primarily through cell-cycle arrest mediated by transcriptional regulation and support its therapeutic relevance in malignancies driven by aberrant CDK9 signaling.

Despite these promising findings, this study had some limitations that must be acknowledged. First, it was based solely on in vitro data, and the in vivo relevance of INTS6 activation remains to be validated in appropriate animal models. Second, the retrospective nature of the clinical dataset and limited number of patients receiving conservative treatment necessitate caution when interpreting the observed prognostic associations. Third, *CTNNB1* mutations have also been identified in a subset of clinical HCC specimens, suggesting that there may be clinical cases in which the therapeutic efficacy of INTS6-saRNA is uncertain [42]. Finally, the precise molecular mechanisms by which INTS6 expression attenuates EMT remain unclear and require further investigation.

## 5. Conclusions

In conclusion, our findings identified INTS6 as a clinically and functionally significant tumor suppressor in HCC. saRNA-mediated upregulation of INTS6 suppresses EMT, impairs cell proliferation, and enhances drug sensitivity, thereby offering a novel gene-regulatory therapeutic strategy. Future in vivo studies and clinical validations are needed to assess the translational potential of INTS6 activation for HCC management. Given that INTS6 downregulation has also been observed in several other malignancies, including breast, lung, and prostate cancers, the therapeutic strategy proposed in this study may have broader applicability beyond HCC.

## Data Availability

The original contributions presented in this study are included in the manuscript/Supplementary Materials. Further inquiries can be directed at the corresponding author.

## Supplementary Materials

The following supporting information can be downloaded at https://www.mdpi.com/article/10.3390/cimb47090733/s1, Table S1: Sequences of saRNA and siRNA; Table S2: Primer sequences for real-time PCR.

## Abbreviations

The following abbreviations are used in this manuscript:

CAR	chimeric antigen receptor
CCK-8	cell-counting Kit-8
CDK9	cyclin-dependent kinase 9
DFS	disease-free survival
EMT	epithelial–mesenchymal transition
GAPDH	glyceraldehyde-3-phosphate dehydrogenase
HCC	hepatocellular carcinoma
IC_50_	half-maximal inhibitory concentration
INTS6	integrator complex subunit 6
LDH	lactate dehydrogenase
lncRNA	long non-coding RNAs
OS	overall survival
PI	propidium iodide
PP2A	protein phosphatase 2A
RNAPII	RNA polymerase II
saRNA	small activating RNA
SD	standard deviation
siRNA	small interfering RNA
TCGA	The Cancer Genome Atlas

## References

[1] Chakraborty E., Sarkar D. (2022). Emerging Therapies for Hepatocellular Carcinoma (HCC). Cancers.

[2] Llovet J.M., Kelley R.K., Villanueva A., Singal A.G., Pikarsky E., Roayaie S., Lencioni R., Koike K., Zucman-Rossi J., Finn R.S. (2021). Hepatocellular carcinoma. Nat. Rev. Dis. Primers..

[3] Villanueva A., Longo D.L. (2019). Hepatocellular Carcinoma. New Engl. J. Med..

[4] Galle P.R., Dufour J.-F., Peck-Radosavljevic M., Trojan J., Vogel A. (2020). Systemic Therapy of Advanced Hepatocellular Carcinoma. Futur. Oncol..

[5] Holohan C., Van Schaeybroeck S., Longley D.B., Johnston P.G. (2013). Cancer drug resistance: An evolving paradigm. Nat. Rev. Cancer.

[6] Shi D., Shi Y., Kaseb A.O., Qi X., Zhang Y., Chi J., Lu Q., Gao H., Jiang H., Wang H. (2020). Chimeric Antigen Receptor-Glypican-3 T-Cell Therapy for Advanced Hepatocellular Carcinoma: Results of Phase I Trials. Clin. Cancer Res..

[7] Mino M., Kanno K., Okimoto K., Sugiyama A., Kishikawa N., Kobayashi T., Ono J., Izuhara K., Kobayashi T., Ohigashi T. (2017). Periostin promotes malignant potential by induction of epithelial-mesenchymal transition in intrahepatic cholangiocarcinoma. Hepatol. Commun..

[8] Negri M., Amatrudo F., Provvisiero D.P., Patalano R., Trinchese G., Cimmino F., de Angelis C., Simeoli C., Auriemma R.S., Mollica M.P. (2025). PER2 expression and cellular localization play a critical role in tumor aggressiveness and drug resistance in an in vitro model of hepatocellular carcinoma. Cancer Drug Resist..

[9] Hu C., Xin Z., Sun X., Hu Y., Zhang C., Yan R., Wang Y., Lu M., Huang J., Du X. (2023). Activation of ACLY by SEC63 deploys metabolic reprogramming to facilitate hepatocellular carcinoma metastasis upon endoplasmic reticulum stress. J. Exp. Clin. Cancer Res..

[10] Giannelli G., Koudelkova P., Dituri F., Mikulits W. (2016). Role of epithelial to mesenchymal transition in hepatocellular carcinoma. J. Hepatol..

[11] Klingenberg M., Matsuda A., Diederichs S., Patel T. (2017). Non-coding RNA in hepatocellular carcinoma: Mechanisms, biomarkers and therapeutic targets. J. Hepatol..

[12] Wan T., Zhang T., Si X., Zhou Y. (2017). Overexpression of EMT-inducing transcription factors as a potential poor prognostic factor for hepatocellular carcinoma in Asian populations: A meta-analysis. Oncotarget.

[13] Wagner E.J., Tong L., Adelman K. (2023). Integrator is a global promoter-proximal termination complex. Mol. Cell.

[14] Welsh S.A., Gardini A. (2022). Genomic regulation of transcription and RNA processing by the multitasking Integrator complex. Nat. Rev. Mol. Cell Biol..

[15] Vervoort S.J., Welsh S.A., Devlin J.R., Barbieri E., Knight D.A., Offley S., Bjelosevic S., Costacurta M., Todorovski I., Kearney C.J. (2021). The PP2A-Integrator-CDK9 axis fine-tunes transcription and can be targeted therapeutically in cancer. Cell.

[16] Sablina A.A., Hahn W.C. (2007). The Role of PP2A A Subunits in Tumor Suppression. Cell Adhes. Migr..

[17] Seshacharyulu P., Pandey P., Datta K., Batra S.K. (2013). Phosphatase: PP2A structural importance, regulation and its aberrant expression in cancer. Cancer Lett..

[18] Xu Y., Liao W., Wang T., Zhang L., Zhang H. (2024). Comprehensive bioinformatics analysis of integrator complex subunits: Expression patterns, immune infiltration, and prognostic signature, validated through experimental approaches in hepatocellular carcinoma. Discov. Oncol..

[19] Lui K.Y., Zhao H., Qiu C., Li C., Zhang Z., Peng H., Fu R., Chen H.-A., Lu M.-Q. (2017). Integrator complex subunit 6 (INTS6) inhibits hepatocellular carcinoma growth by Wnt pathway and serve as a prognostic marker. BMC Cancer.

[20] Tong H., Liu X., Li T., Qiu W., Peng C., Shen B., Zhu Z. (2019). INTS8 accelerates the epithelial-to-mesenchymal transition in hepatocellular carcinoma by upregulating the TGF-β signaling pathway. Cancer Manag. Res..

[21] Jia Y., Cheng Z., Bharath S.R., Sun Q., Su N., Huang J., Song H. (2021). Crystal structure of the INTS3/INTS6 complex reveals the functional importance of INTS3 dimerization in DSB repair. Cell Discov..

[22] Yoon S., Rossi J.J. (2018). Therapeutic Potential of Small Activating RNAs (saRNAs) in Human Cancers. Curr. Pharm. Biotechnol..

[23] Tan C.P., Sinigaglia L., Gomez V., Nicholls J., Habib N.A. (2021). RNA Activation—A Novel Approach to Therapeutically Upregulate Gene Transcription. Molecules.

[24] Lachenmayer A., Alsinet C., Savic R., Cabellos L., Toffanin S., Hoshida Y., Villanueva A., Minguez B., Newell P., Tsai H.-W. (2012). Wnt-Pathway Activation in Two Molecular Classes of Hepatocellular Carcinoma and Experimental Modulation by Sorafenib. Clin. Cancer Res..

[25] Chen H., Shen H.-X., Lin Y.-W., Mao Y.-Q., Liu B., Xie L.-P. (2018). Small RNA-induced INTS6 gene up-regulation suppresses castration-resistant prostate cancer cells by regulating β-catenin signaling. Cell Cycle.

[26] Wang J., Place R.F., Portnoy V., Huang V., Kang M.R., Kosaka M., Ho M.K.C., Li L.-C. (2015). Inducing gene expression by targeting promoter sequences using small activating RNAs. J. Biol. Methods.

[27] Mir N., Jayachandran A., Dhungel B., Shrestha R., Steel J.C. (2017). Epithelial-to-Mesenchymal Transition: A Mediator of Sorafenib Resistance in Advanced Hepatocellular Carcinoma. Curr. Cancer Drug Targets.

[28] Kudo M., Finn R.S., Qin S., Han K.-H., Ikeda K., Piscaglia F., Baron A., Park J.-W., Han G., Jassem J. (2018). Lenvatinib versus sorafenib in first-line treatment of patients with unresectable hepatocellular carcinoma: A randomised phase 3 non-inferiority trial. Lancet.

[29] Finn R.S., Ryoo B.-Y., Merle P., Kudo M., Bouattour M., Lim H.Y., Breder V., Edeline J., Chao Y., Ogasawara S. (2020). Pembrolizumab As Second-Line Therapy in Patients With Advanced Hepatocellular Carcinoma in KEYNOTE-240: A Randomized, Double-Blind, Phase III Trial. J. Clin. Oncol..

[30] Finn R.S., Qin S., Ikeda M., Galle P.R., Ducreux M., Kim T.-Y., Kudo M., Breder V., Merle P., Kaseb A.O. (2020). Atezolizumab plus Bevacizumab in Unresectable Hepatocellular Carcinoma. N. Engl. J. Med..

[31] Li K., Lin S.Y., Brunicardi F.C., Seu P. (2003). Use of RNA interference to target cyclin E-overexpressing hepatocellular carcinoma. Cancer Res..

[32] Liu J.-Y., Chiang T., Liu C.-H., Chern G.-G., Lin T.-T., Gao D.-Y., Chen Y. (2015). Delivery of siRNA Using CXCR4-targeted Nanoparticles Modulates Tumor Microenvironment and Achieves a Potent Antitumor Response in Liver Cancer. Mol. Ther..

[33] Bogorad R.L., Yin H., Zeigerer A., Nonaka H., Ruda V.M., Zerial M., Anderson D.G., Koteliansky V. (2014). Nanoparticle-formulated siRNA targeting integrins inhibits hepatocellular carcinoma progression in mice. Nat. Commun..

[34] Reebye V., Sætrom P., Mintz P.J., Huang K.-W., Swiderski P., Peng L., Liu C., Liu X., Lindkær-Jensen S., Zacharoulis D. (2013). Novel RNA oligonucleotide improves liver function and inhibits liver carcinogenesis in vivo. Hepatology.

[35] Voutila J., Reebye V., Roberts T.C., Protopapa P., Andrikakou P., Blakey D.C., Habib R., Huber H., Saetrom P., Rossi J.J. (2017). Development and Mechanism of Small Activating RNA Targeting CEBPA, a Novel Therapeutic in Clinical Trials for Liver Cancer. Mol. Ther..

[36] Sarker D., Plummer R., Meyer T., Sodergren M.H., Basu B., Chee C.E., Huang K.-W., Palmer D.H., Ma Y.T., Evans T.J. (2020). MTL-CEBPA, a Small Activating RNA Therapeutic Upregulating C/EBP-α, in Patients with Advanced Liver Cancer: A First-in-Human, Multicenter, Open-Label, Phase I Trial. Clin. Cancer Res..

[37] Peng H., Ishida M., Li L., Saito A., Kamiya A., Hamilton J.P., Fu R., Olaru A.V., An F., Popescu I. (2015). Pseudogene INTS6P1 regulates its cognate gene INTS6 through competitive binding of miR-17-5p in hepatocellular carcinoma. Oncotarget.

[38] Suo J., Medina D., Herrera S., Zheng Z.-Y., Jin L., Chamness G.C., Contreras A., Gutierrez C., Hilsenbeck S., Umar A. (2015). Int6 reduction activates stromal fibroblasts to enhance transforming activity in breast epithelial cells. Cell Biosci..

[39] Federico A., Rienzo M., Abbondanza C., Costa V., Ciccodicola A., Casamassimi A. (2017). Pan-Cancer Mutational and Transcriptional Analysis of the Integrator Complex. Int. J. Mol. Sci..

[40] Galle E., Thienpont B., Cappuyns S., Venken T., Busschaert P., Van Haele M., Van Cutsem E., Roskams T., van Pelt J., Verslype C. (2020). DNA methylation-driven EMT is a common mechanism of resistance to various therapeutic agents in cancer. Clin. Epigenetics.

[41] Filleur S., Hirsch J., Wille A., Schön M., Sell C., Shearer M.H., Nelius T., Wieland I. (2009). INTS6/DICE1 inhibits growth of human androgen-independent prostate cancer cells by altering the cell cycle profile and Wnt signaling. Cancer Cell Int..

[42] Oversoe S.K., Clement M.S., Weber B., Grønbæk H., Hamilton-Dutoit S.J., Sorensen B.S., Kelsen J. (2021). Combining tissue and circulating tumor DNA increases the detection rate of a CTNNB1 mutation in hepatocellular carcinoma. BMC Cancer.

